# Supplementary material for: Streptococcus pyogenes NAD+-Glycohydrolase Reduces Skeletal Muscle βNAD+ Levels Independently of Streptolysin O
Source: Microorganisms. 2022 Jul 21;10(7):1476. doi: 10.3390/microorganisms10071476 (PMC9322677; doi:10.3390/microorganisms10071476)
Supplement: Supplementary file 1 [file microorganisms-10-01476-s001.zip › microorganisms-1820119-supplementary.pdf]

## SUPPORTING INFORMATION

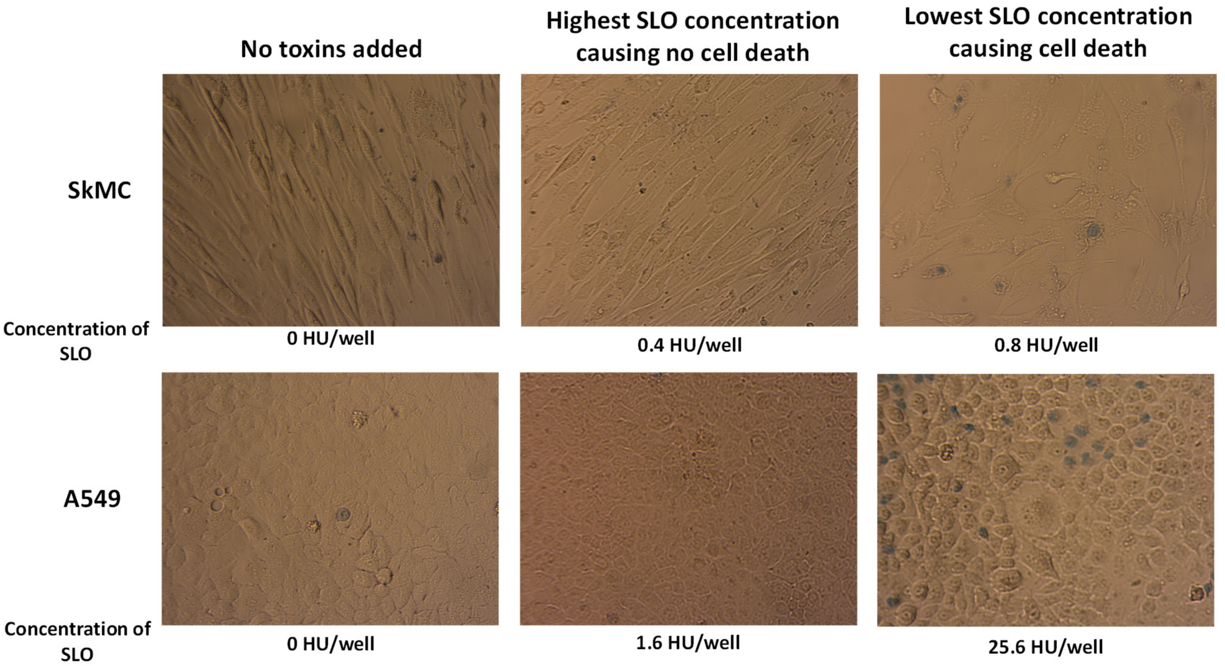


**Supplementary Figure S1. Toxin concentrations used in this study did not induce cell death after 2 hours of toxin exposure**. SkMCs or A549s were incubated with 0.03 Units of SPN (the concentration in ~1:8 dilution of culture supernatant and the concentration of purified SPN used in this study) plus 0 to 25.6 HU/well of SLO at 37°C for 2 hours. SLO concentrations were based on the amount used in recombinant studies (0 - 1.6 HU/well) and higher concentrations were used to illustrate cell death. After toxin treatment, media was aspirated and stained with trypan blue. After 3 minutes cells were washed and visualized using phase contrast. Images represent one of 5 in the field after no treatment, the highest concentration of SLO in which no death trypan blue stain was seen and the lowest concentration of SLO that caused cell death where many cells were stained with trypan blue. SLO concentrations are indicated below each image.

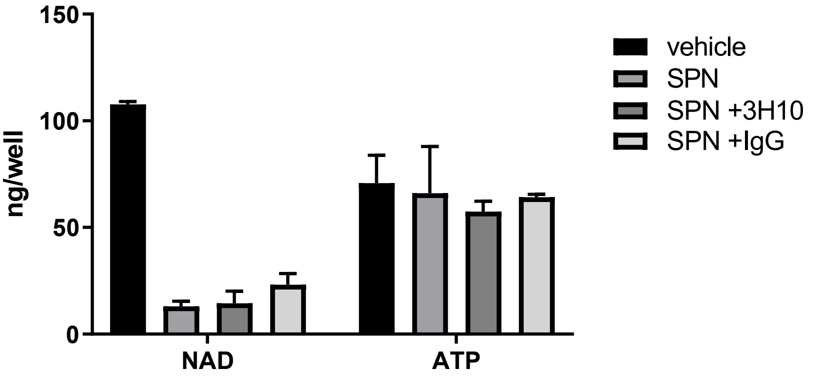


**Supplementary Figure S2. SPN-induced NAD+ depletion is not due to contaminating SLO**. Purified SPN was incubated with either 10 µg/mL neutralizing anti-SLO antibody (3H10) or an isotype matched control antibody for 30 min at 37°C. The vehicle control was similarly treated. Treatments were then added to SkMCs and NAD+ measured by cycling assay after 1 hour of toxin exposure. Data are from a single experiment done in duplicate. Error bars represent SD.
